# Telomere Length and Telomerase Activity as Potential Biomarkers for Gastrointestinal Cancer

**DOI:** 10.3390/cancers16193370

**Published:** 2024-10-01

**Authors:** Christina Loukopoulou, Taxiarchis Nikolouzakis, Ioannis Koliarakis, Elena Vakonaki, John Tsiaoussis

**Affiliations:** 1 Department of Anatomy, School of Medicine, University of Crete, 71003 Heraklion, Greece; cloukopo@exseed.ed.ac.uk (C.L.); medp2011836@med.uoc.gr (T.N.); medp2011931@med.uoc.gr (I.K.); 2 Department of Forensic Sciences and Toxicology, School of Medicine, University of Crete, 71003 Heraklion, Greece; evakonaki@med.uoc.gr

**Keywords:** telomeres, telomere length, telomerase, telomerase activity, biomarker, gastrointestinal cancer

## Abstract

**Simple Summary:**

Cancer affects millions worldwide, with gastrointestinal cancers being notably common. This review explores the potential of telomeres and telomerase as biomarkers for diagnosing and predicting these cancers. Telomeres are protective caps at chromosome ends that shorten with cell division, while telomerase helps extend them. Changes in these markers could indicate cancer risk and progression. Despite efforts to prevent cancer through lifestyle changes, gastrointestinal cancers such as colorectal and gastric cancer remain prevalent. Current detection methods like endoscopy are invasive, driving interest in non-invasive alternatives. By consolidating research on telomere length and telomerase activity, this review aims to highlight how these biomarkers could enhance early diagnosis and monitoring. Understanding these mechanisms better could lead to improved diagnostic tools and therapeutic strategies, potentially refining how gastrointestinal cancers are managed and improving patient outcomes.

**Abstract:**

Gastrointestinal (GI) cancers, such as colorectal and gastric cancers, pose significant global health challenges due to their high rates of incidence and mortality. Even with advancements in treatment and early detection, many patients still face poor outcomes, highlighting the critical need for new biomarkers and therapeutic targets. Telomere length (TL) and telomerase activity (TA) have gained attention in this context. Telomeres, protective nucleotide sequences at chromosome ends, shorten with each cell division, leading to cellular aging. Telomerase, a ribonucleoprotein enzyme, counteracts this shortening by adding telomeric repeats, a process tightly regulated in normal cells but often dysregulated in cancer. This review critically evaluates the role of TL and TA in the pathogenesis of GI cancers, examining their potential as diagnostic, prognostic, and predictive biomarkers. It explores how alterations in telomere biology contribute to the initiation and progression of GI tumors and assesses the therapeutic implications of targeting telomerase. By integrating findings from diverse studies, this review aims to elucidate the intricate relationship between telomere dynamics and gastrointestinal carcinogenesis, offering insights into how TL and TA could be leveraged to enhance the early detection, treatment, and prognosis of GI cancers.

## 1. Introduction

In 2022, an estimated of 20 million new cancer cases were documented worldwide, with 26% of which affecting the gastrointestinal (GI) tract. In detail, colorectal cancer (CRC) was the most prevalent malignancy (9.6%), followed by gastric cancer (6.8%) [1,2]. However, despite great efforts from the World Health Organization and national scientific societies to decrease their incidence, malignancies of the GI tract seem to maintain their incidence with CRC being steadily placed in the second place of the most common type of cancer in both sexes. An abundance of evidence suggests that more than half of all the malignancies of the GI tract are attributed to modifiable risk factors such as unhealthy diet habits (high fat, high sugar, low fiber), increased alcohol consumption, smoking, obesity, and low physical activity [1,2]. In fact, these risk factors are the key targets of primary prevention strategies that aim to decrease the carcinogenic risks that modern societies face.

Unfortunately, to a certain extent, these risk factors have become part of the Western lifestyle that more and more people follow across the globe and this is the reason why primary prevention has failed to achieve more significant results [1,2,3,4]. Secondary prevention aims to detect premalignant lesions or early-state malignancies and treat them in situ. To do so, endoscopy of the upper and lower GI tract has become the modality of choice as it offers the advantage of diagnosis and treatment in the same session. However, evidence suggests that adherence to endoscopy protocols is significantly lower than expected partly due to the invasive nature of the test. For this reason, non-invasive tests have been introduced to assist in an early diagnosis while also providing valuable information regarding the prognosis and prediction in cases where a cancer has been diagnosed or a specific treatment has been chosen [5,6]. One major step in improving early diagnosis, prognosis, and prediction in regard to cancer is the introduction of biomarkers in everyday clinical practice [7]. A biomarker is a biological molecule found in body fluids or tissues that indicates a normal or abnormal process, condition, or disease [6]. Tumor biomarkers are substances that can be detected in tissues or body fluids such as blood, urine, stool, and saliva [6]. They can generally be synthesized directly by the tumor or by the body in response to the presence of cancer [6,8]. Two promising biomarkers with diagnostic, prognostic, or predictive significance in GI tract malignancies are telomere length (TL) and telomerase activity (TA), both of which are important regulators of normal cell function while also playing an important role in carcinogenesis. Overall, the aim of this review paper is to consolidate the findings from published studies on the relationship between TL, TA, and their roles as diagnostic, prognostic, and predictive biomarkers in gastrointestinal cancer.

### 1.1. Telomeres

Telomeres, consisting of repetitive nucleotide sequences (5′-TTAGGG-3′), are special DNA structures found at the ends of chromosomes which are made up of repeated hexameric DNA sequences (5′-TTAGGG-3′) and a protein complex called shelterin, formed by six proteins: TRF1, TRF2, TIN2, POT1, TPP1, and RAP1 [9,10]. Telomeres aid in the prevention of degradation and help distinguish chromosome ends from double-strand breaks [11,12]. Working along with shelterin proteins, they play a critical role in maintaining chromosome stability and protecting them from attrition [13]. Telomeric DNA ends with a G-rich single-strand overhang ranging from 50 to 300 nucleotides, folding back to create a “T-loop”, encompassing telomeres and shelterins [11]. T-loops play a crucial role in safeguarding telomeres by preventing DNA repair mechanisms from mistakenly identifying them as double-stranded DNA breaks. With each mitotic cell division, there is a strand of telomere DNA at the chromosome end which lags behind. This cannot be fully replicated by DNA polymerases, resulting in an annual rate of telomere shortening by ~20–50 bp, which ultimately leads to cell proliferation arrest and senescence [14]. This phenomenon known as the ‘end replication problem’ causes a gradual reduction in average telomere length with the rate accelerating after the age of 50 [15,16]. Telomere shortening is influenced by genetic factors like inherited telomere length and telomerase activity, lifestyle factors such as smoking, diet, and physical activity, and environmental factors including stress and exposure to pollutants. Chronic inflammation and diseases can also accelerate telomere shortening [17,18,19,20,21]. Therefore, average TL is suggested as a marker of the ‘biological age’ of cells and the organism as a whole [16].

With each cell division, telomeres become shorter as the chromosome’s 3′ end cannot be fully replicated [11]. However, cancer cells can reactivate telomerase, allowing them to divide indefinitely [22]. Telomerase-expressed reverse transcriptase (TERT) is silenced to prevent indefinite cell replication, leading to apoptosis or cell senescence. Telomere homeostasis depends on TL and the concentration of active telomerase. Shelterin complex proteins, which typically include TRF1, TRF2, RAP1, POT1, TIN2, and TPP1, can regulate telomere homeostasis by inhibiting telomerase action. TA is restricted to the S phase of the cell cycle, and not all telomeres undergo lengthening after DNA replication—only those prioritized based on their telomere lengths [23].

Genome instability is widely recognized as a key characteristic of carcinogenesis and is considered key in cancer development.

In the accepted model of carcinogenesis, telomeres in somatic cells gradually shorten with every cell division [24]. After roughly 50 to 60 cell divisions, shortened telomeres cause chromosomal instability, leading to replicative senescence through the activation of p53. This process is triggered by DNA damage linked to telomere shortening [24].

A decrease in telomere length with age can contribute to genetic instability and raise the likelihood of cancer. TL measured in peripheral blood of individuals in their seventies is predictive of survival, with the strongest association found between TL and mortality from heart disease and infections. It is said that telomere dysfunction occurs early in the development of cancer in ulcerative colitis, leading to chromosomal instability and the formation of anaphase bridges [25,26]. This process may expedite the molecular progression of carcinogenesis by promoting chromosomal instability in cells [27,28].

### 1.2. Telomerase

**Figure 1:**
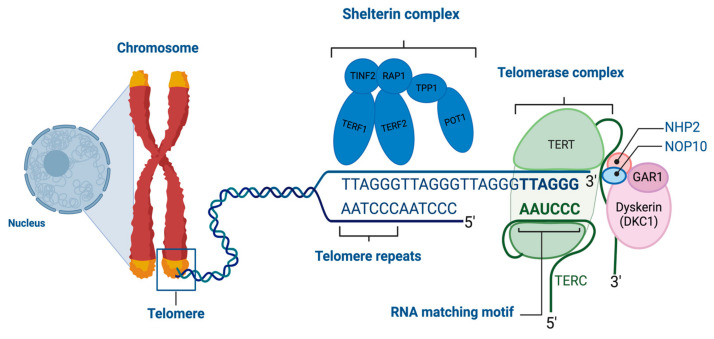
Telomere lengthening by telomerase. “Created with Biorender.com”.

The ribonucleoprotein enzyme telomerase acts to maintain telomere length by employing an RNA template in its reverse-transcriptase function to synthesize telomeric DNA repeats [27]. Telomerase consists of telomerase reverse transcriptase (TERT) and telomeric RNA component (TERC) [12,22,28] (Figure 1).

Although TERT is mostly found in the nucleus, a portion (10–20%) is located in the mitochondria. TERT’s non-telomeric roles include reducing reactive oxygen species (ROS), DNA damage, and apoptosis. Recent research has shown that TERC enters mitochondria, is processed into a shorter form (TERC-53), and then returns to the cytosol. Damage to mitochondria, such as that caused by mitochondrial ROS, can lead to telomere dysfunction. Additionally, ROS from abnormal mitochondrial activity can cause single-strand breaks in telomeres, contributing to their dysfunction. Dysfunctional telomeres suppress PGCα/β in a p53-dependent manner, reducing ATP production, increasing ROS levels, and impairing metabolic functions like gluconeogenesis. This indicates a link between telomeres and mitochondria. Therefore, identifying more mediators in this interaction is essential for understanding the molecular mechanisms underlying carcinogenesis and cancer progression [10,29].

Human telomerase RNA is consistently present across various tissues, making human telomerase reverse transcriptase (hTERT) the critical factor controlling telomere activity. In some cancers, the loss of telomeres can be counterbalanced by the alternative lengthening of telomeres (ALT) pathway, which operates independently of telomerase [12,30]. In the absence of telomerase, the alternative lengthening of telomeres (ALT) pathway relies on homologous recombination for DNA replication to upkeep cellular longevity. While telomerase is present in 85–95% of cancer cells, approximately 5–15% of these cells use the ALT mechanism to sustain their telomeres [24,31,32].

Cellular senescence functions as a crucial safeguard against cancer by inhibiting cells from achieving immortality. In response, cancer cells often elevate their TA to mitigate the natural shortening of telomeres during cell division. This increase is driven by varying levels of hTERT gene expression observed between cancerous and normal cells [28]. It has been observed that telomerase is active in about 90% of human tumors, crucially maintaining telomere lengths and promoting tumor growth [29,33]. Thus, TA will be higher in tumors compared to healthy tissue. An early study by Shay and Bacchetti revealed that increased TA is generally reported in more than 85% of cancer cells [31]. It is intriguing to consider that cells with severely shortened telomeres might activate telomerase under specific conditions to evade cellular senescence, thereby facilitating the development of cancer [34,35].

Nevertheless, it is essential to note that the presence of the longest telomeres does not necessarily correspond to the highest TA. Upon activation, telomerase can maintain telomeres at varying lengths. Crucially, the relationship between TL and TA is indicative of the extent of cancer cell infiltration [36,37].

## 2. Telomere Length and Telomerase Activity in Gastrointestinal Cancers

Telomeres serve as crucial chromosome protectors, essential for preserving genomic stability. These DNA–protein complexes consist of repetitive “5′-TTAGGG-3′” sequences and an associated terminal protein complex. Over time, telomeres progressively shorten, leading to chromosomal instability in normal somatic cells. This shortening is linked to various aging-related diseases, including gastrointestinal cancer [11,35,37]. However, the relationship between TL and cancer risk remains a subject of debate, with studies yielding conflicting results [38].

Pooley et al. observed that significant telomere shortening primarily takes place after a cancer diagnosis, rather than before or during its development [16]. Oxidative stress accelerates telomere shortening by creating single-strand DNA breaks from oxidative or alkylative damage, which are inadequately repaired in the telomeres [39,40,41]. Previous research has shown that people with shorter average telomere lengths might be at a higher risk of dying from various diseases [16].

In 2016, Zhu and colleagues conducted a comprehensive review of studies examining the link between telomere length and cancer risk [42]. The analysis, which included data from 23,379 cancer patients and 68,792 controls across 51 studies, identified a significant correlation between telomere length and gastrointestinal and head and neck cancers. However, no significant connection was found between telomere length and overall cancer risk [42].

**Figure 2:**
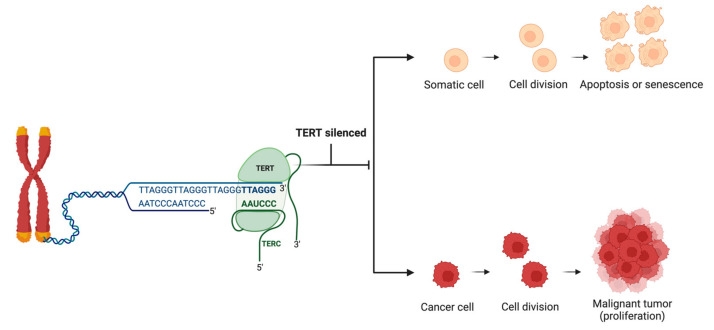
Telomerase activity in healthy somatic cells vs. cancer cells. Telomerase, an enzyme essential for maintaining telomere length, comprises the TERT subunit, which acts as a reverse transcriptase, and the TERC subunit, which provides a template for telomere extension. In healthy somatic cells, TERT is typically suppressed to prevent unchecked cell division, leading to cellular aging and programmed cell death. In contrast, cancer cells often reactivate telomerase, enabling continuous proliferation and the accumulation of genetic mutations. “Created with Biorender.com”.

Telomerase is commonly activated in cancers to lengthen telomeres (Figure 2). Barthel et al. analyzed telomere lengths in 18,430 samples from 31 types of cancer using data from The Cancer Genome Atlas (TCGA). Their findings revealed that 70% of these cancers had shorter telomeres compared to normal samples, which was associated with increased telomerase activity [43]. In the remaining 30% of cancer types, telomere length was observed to be the same as control or longer due to suspected alternative lengthening of telomeres (ALT) activity [24,43,44]. Conversely, TA is significantly increased in the majority of cancers studied across various malignancies, including colorectal cancer [45,46,47] and pancreatic cancer [45,46,48]. The distinct contrast in appearance between normal or benign tissues and malignant tissues indicates that telomerase could serve as a universal biomarker for cancer diagnosis and prognosis.

Below, Table 1 provides a collective summary of studies investigating TL and TA in gastrointestinal cancers and their respective bibliographic references.

### 2.1. Esophageal Cancer

The reduction in telomere length begins early in the process of esophageal cancer development, while short telomeres on chromosomes 17p and 12q have been linked to a higher risk of esophageal cancer [59]. Wennerström et al. conducted a study to explore whether individuals with gastroesophageal reflux disease, a known risk factor for esophageal adenocarcinoma, had shorter telomeres in their white blood cells [60]. The researchers found no increased prevalence of either shorter or longer telomeres in individuals with esophageal cancer.

Valdes et al. and O’Sullivan et al. observed a direct correlation between telomere length and advancing age [19,26]. This finding was reinforced by research from Xing and colleagues, who investigated chromosome-specific telomere lengths on chromosomes 17p, 12q, 2p, and 11q in relation to esophageal cancer [59].

In research conducted by Risques et al., telomere length in the blood of Barrett’s esophagus patients was found to be a predictor of their likelihood of developing esophageal adenocarcinoma [39]. This association held true even when other factors like gender, age, and smoking were considered. Patients with the shortest leukocyte telomeres had the greatest risk, especially if they were smokers. This highlights the role of short telomeres as a strong indicator of cancer risk, which can be influenced by environmental factors like smoking and oxidative stress [41].

In contrast, Lv et al. reported that longer telomeres are linked to a worse prognosis, establishing them as a standalone negative prognostic factor for esophageal cancer patients [61]. This finding supports the data from Li et al. indicating that inhibition of hTERT expression is able to restrain the migration and invasion abilities of malignant cells in the case of esophageal squamous cell carcinoma [62]. In a study by Li et al., it was found that there is increased telomerase activity in esophageal squamous cell carcinoma (ΕSCC) [103]. Ikeguchi et al. and Takubo et al. found that telomerase activity was present in both cancerous tissues and normal esophageal mucosa, indicating that telomerase activity might not serve as a dependable biomarker for esophageal cancer detection [63,64].

Pursuing this further, Mitsui et al. observed in their study that telomerase activity was found in 79.6% of tumors and 59.3% of normal tissues, with significantly higher levels in the tumors [65]. Tumors with extensive blood vessel invasion exhibited increased telomerase activity. Additionally, tumors that responded well to preoperative chemotherapy had notably lower telomerase activity compared to those that did not respond. In an esophageal cancer cell line, treatment with the chemotherapy drug 5-FU led to a reduction in hTERT mRNA expression, resulting in decreased telomerase activity. These findings indicate that telomerase activity is linked to tumor invasion and chemotherapy response in esophageal cancer.

### 2.2. Gastric Cancer

In 2018, gastric cancer ranked as the second most prevalent cancer in the gastrointestinal tract, with more than one million new cases and nearly 800,000 deaths [4].

Based on a long-term study conducted by Shi et al. involving a high population of gastric cancer patients, the authors reported that individuals with shorter telomeres in cell-free DNA faced a heightened risk of gastric cancer progression [51]. Furthermore, shortened telomeres could be detected over a three-year period prior to the diagnosis of gastric cancer.

In research conducted by Wang et al., involving Chinese participants with an average age of 67, both extremely short and long telomeres were linked to a higher risk of gastric cancer, with increased risks of 63% and 55%, respectively [52]. These findings are consistent with observations made by Hou et al. and Wentzensen et al. [11,50]. The association between telomere length and cancer risk may be due to short telomeres causing chromosomal instability and initiating cancer, while long telomeres could lead to excessive cell division and genetic abnormalities, thereby increasing cancer risk [52,53].

In another study, the authors reported shorter telomeres in gastric cancer tumor cells at an early stage than those in the surrounding non-cancerous tissue cells [104]. Telomere shortening seems to initiate gastric cancer, while the extension or maintenance of telomere length via telomerase reactivation is crucial for the cancer’s invasion and progression. Liu et al. supported these findings by showing that patients with gastric adenocarcinoma had significantly shorter leukocyte telomeres compared to controls. Additionally, progressive telomere shortening was associated with an increased risk of developing the disease [38].

Katayama et al. studied telomerase activity and telomere length in gastric and colorectal cancers [55]. They found that TA was present in 8% of gastric polyps and 22% of colorectal polyps. In contrast, TA was significantly more common in gastric cancer (70%) and colorectal cancer (81%), with *p*-values below 0.0003 and 0.0001, respectively.

In a similar study, Rathi et al. analyzed human telomerase RNA (hTR) expression in gastric cancer through in situ hybridization on tissue samples [56]. They found that hTR levels were low in normal gastric mucosa, chronic peptic ulcers, and hyperplastic polyps. Tubular adenomas exhibited weak but more widespread hTR expression. In contrast, gastric carcinomas showed moderate to high levels of hTR, with expression intensity increasing as the cancer progressed. These findings suggest that telomerase upregulation occurs early in gastric cancer development and could serve as an important marker for early detection.

Another study by Hu et al. examined the importance of telomerase activity in gastric carcinoma tissues and peritoneal washings [57]. Telomerase activity was present in 89.1% of gastric carcinomas and 47.8% of peritoneal washings, and it was linked to factors such as histological grade, invasion depth, serosal invasion, and peritoneal metastasis. The detection rate in peritoneal washings was significantly higher than those found by cytology (26.1%) and cancer antigen 125 (CA125) (34.8%). The authors proposed that telomerase activity could be an effective diagnostic marker for gastric carcinoma and that identifying it in peritoneal washings via TRAP-ELISA may aid in detecting early peritoneal dissemination in gastric cancer patients. These results were in agreement with findings reported by Svinareva et al. investigating telomerase activity in tissue specimens from patients with gastric adenocarcinomas and gastric lymphoma using a modified TRAP assay [58]. Telomerase activity was present in 16 of 18 (89%) patients with gastric adenocarcinomas and in 1 patient with gastric lymphoma, but not in the control patient with non-cancerous gastric tissue. The majority of the samples (88%) showed “high” or “very high” levels of telomerase activity. These findings suggest a strong correlation between telomerase activity and malignancy, proposing its potential as a diagnostic marker for gastric cancer [58].

Furthermore, Pascua et al. explored how telomere function affects the prognosis of gastric cancer with different levels of microsatellite instability (MSI) [105]. Their study revealed that patients with high microsatellite instability had significantly better outcomes compared to those with microsatellite-stable or low-microsatellite-instability tumors. They also observed that tumors with the shortest telomeres were associated with a worse prognosis. Similarly, Mushtaq et al. found that shorter telomeres and elevated hTERT expression were related to the progression of gastric cancer [54].

### 2.3. Colorectal Cancer

Numerous cancers associated with shortened telomeres, such as bladder and gastric cancers, are often linked to chronic inflammation or carcinogenic exposures like smoking, in the case of bladder and lung cancers [11]. Chronic inflammation, which is a recognized risk factor for cancers like esophageal, bladder, and gastric cancers, leads to an increased turnover of granulocytes [11]. Since telomeres shorten with each cell division, this accelerated turnover can lead to shorter telomeres in granulocytes and a reduced overall length of leukocyte telomeres [11,106]. Certain cytokines have the ability to activate telomerase, potentially mitigating telomere shortening to some extent. Ulcerative colitis illustrates the complex relationship between inflammation, cancer risk, and telomere dynamics [11,107]. Individuals with this condition face a higher risk of colon cancer, especially those with increased chromosomal instability and shorter telomeres [11,108].

Research indicates a possible link between intestinal microbiota and TL, especially in the context of aging and oxidative stress. This connection highlights the potential of using TL as a diagnostic and prognostic marker, as well as a target for novel anti-cancer therapies, due to its role in colorectal cancer development. Telomeres shorten with each cell division and this process speeds up with age, leading to an increase in senescent cells that release inflammatory cytokines and contribute to age-related diseases [109]. Factors such as poor diet, smoking, and inactivity raise reactive oxygen species (ROS) levels, accelerating telomere shortening [110]. Additionally, aging-related mitochondrial dysfunction, which boosts ROS production, further aggravates telomere damage [109].

Oxidative stress speeds up telomere shortening by damaging telomeric DNA, including single-strand breaks and base alterations, with guanine being particularly vulnerable [109,111]. Antioxidants like peroxiredoxin 1, which protect telomeres, decrease with age, leading to further telomere damage [111]. Intestinal microbiota can influence oxidative stress and inflammation [109,112]. Certain bacteria associated with inflammatory bowel disease and colorectal cancer, including *enterotoxigenic B. fragilis*, *adherent-invasive E. coli*, and *Fusobacterium nucleatum* among others, increase oxidative stress [113]. Gut bacteria also impact oxidative stress by modulating mitochondrial activity and producing short-chain fatty acids like acetate and butyrate, which are linked to reduced oxidative stress and inflammation [114,115].

Recent studies have indicated that changes in TL independently correlate with the progression and prognosis of colorectal cancer [26,66,67,68,69,70,71,72,73]. However, the abovementioned studies measured telomere length in colonocytes taken from paired samples of cancerous and non-cancerous tissues within the same individuals, compared to peripheral blood leukocyte telomere lengths as described in a study conducted by Zee et al. [74]. Collectively, these findings indicate that telomere behavior in colonocytes varies from that observed in other tissues, including peripheral blood leukocytes.

When considering tumor location, rectal cancers generally have a poorer prognosis compared to colon cancers, and clinical treatments vary accordingly [75,76]. Previous findings have shown that TL differs based on tumor location, with rectal cancers typically exhibiting longer telomeres [76,77]. These results align with the findings of Zöchmeister et al. and Peacock et al., who reported that telomeres were significantly longer in colorectal cancer patients compared to controls [78,83]. Additionally, Kibriya et al. found that among 165 colorectal cancer patients, telomere shortening was more pronounced in lower-grade cancers and in those with microsatellite instability [30]. Other research indicates that microsatellite instability is found in approximately 15% of colorectal cancers, whereas chromosomal instability with stable microsatellites is often linked to TP53 gene mutations [30,79,80].

In Kroupa et al.’s study, telomeres were shorter in tumor tissues compared to adjacent mucosa in 74% of colorectal cancer patients [81]. Shorter telomeres were linked to tumors in the proximal colon, microsatellite instability, mucinous histology, and earlier TNM stages. Similarly, metastatic liver tissues showed reduced telomere length compared to adjacent healthy liver tissue. Patients with a smaller difference in telomere length between tumor and adjacent tissue had better survival outcomes. These findings indicate that telomere length variations, depending on tumor location and characteristics, may influence prognosis and treatment approaches.

In a later study by the same authors expanding the focal point to metastatic colorectal cancer lesions, it was reported that most primary tumors showed shorter telomeres compared to adjacent non-cancerous tissue, indicating a potential role in malignant transformation [82]. Metachronous liver metastases showed shorter telomeres than synchronous ones, with the shortest telomeres found in proximal colon tumors. Neoadjuvant chemoradiotherapy notably shortened telomeres in rectal tumors and nearby tissues. A higher tumor-to-mucosa telomere length ratio was associated with better overall survival. These findings underscore the significance of telomere dynamics in colorectal cancer development.

Ye et al. examined telomere length in colorectal cancer and adenoma cells using quantitative fluorescent in situ hybridization (Q-FISH) [116]. The results indicated that telomere fluorescent intensity units (TFUs) were significantly lower in carcinoma and adenoma cells compared to cancer-associated fibroblasts (CAF), with adenoma cells exhibiting the shortest telomeres. In carcinoma cells, reduced telomere fluorescent intensity units (TFUs) and relative telomere length (RTL) were associated with distant metastases and poorer overall and disease-free survival. These findings suggest that telomere shortening might happen early in colorectal cancer progression and could serve as a prognostic indicator for the disease.

Telomerase has been recognized as a crucial predictor of overall survival in colorectal cancer patients, with those having higher TERT levels showing markedly worse survival rates compared to those with lower TERT levels [79].

Engelhardt et al. examined telomerase levels in various colon tissues, finding that while telomerase is generally low in non-cancerous cells, it was notably higher in colorectal cancer samples. Their study analyzed 130 frozen specimens, including cancerous tissues, adjacent normal tissues, polyps, and colitis. They detected moderate to high telomerase activity in 90% of colorectal tumors and weak activity in the remaining 10%. Normal colon tissues showed no telomerase activity, and polyps and colitis had significantly lower levels than cancerous tissues. Higher telomerase activity was associated with advanced-stage tumors compared to early-stage ones [71]. In a separate study by Garcia-Aranda et al., 91 primary colorectal cancers and their normal counterparts were assessed for telomere length and telomerase activity. This research found that 81.3% of tumors had telomerase activity, and telomeres were shorter in cancer tissues compared to normal ones (*p* = 0.02). Tumors with shorter telomeres also had higher levels of TRF1 [67].

### 2.4. Liver Cancer

Telomere shortening is a feature observed in intrahepatic cholangiocarcinoma (iCCA), akin to what is seen in hepatocellular carcinoma (HCC). In a healthy liver, cholangiocytes typically have longer telomeres compared to other liver cells, and telomere shortening related to aging is not prominent in the absence of liver disease. However, telomere shortening becomes evident early in the progression of biliary tract carcinoma, beginning with inflammation of the biliary tract and continuing through metaplasia, dysplasia, and carcinoma [116]. Telomerase activity is present in a majority of both HCC and iCCA cases, indicating its importance in telomere maintenance and tumor development. Despite this, the mechanisms behind telomerase reactivation differ between HCC and iCCA. TERT promoter mutations are commonly found in HCC but are rare in iCCA, suggesting that other mechanisms like epigenetic or transcriptional regulation might be more significant in iCCA. These differences highlight the need for further research to identify potential therapeutic targets specific to iCCA [116].

Research has shown that telomere shortening is common in chronic liver disease [84,117]. The impact of telomere length on liver cancer risk varies depending on the cancer’s stage. Reduced telomeres can promote liver cancer development [41,85], and the risk of hepatocellular carcinoma (HCC) increases notably during cirrhosis, which is associated with shorter hepatocyte telomeres. Interestingly, while telomeres are generally shorter in HCC patients compared to healthy controls, those with advanced HCC often have longer telomeres [22,86,87].

In normal human liver tissue, there is no significant telomerase activity [88]. In contrast, more than 80% of human HCC exhibit increased level of TA primarily due to the re-expression of hTERT [89,90]. Moreover, increased TA in HCC or surrounding healthy tissue has been identified as an indicator of poor prognosis since it correlates with post-operative recurrence and poor survival [91,92].

### 2.5. Pancreatic Cancer

Telomere length and telomerase activity play significant roles in the development and progression of pancreatic cancer. Unlike colorectal cancer, where shortened telomeres are prevalent, Duell’s research suggests that reduced telomere length might increase the risk of pancreatic cancer and its progression [93]. Conversely, Lynch et al. found that longer telomeres were linked to a higher risk of pancreatic cancer [94], a conclusion also supported by Luu et al. and Campa et al. [95,96]. Additionally, Skinner’s extensive case-control study demonstrated a clear connection between shorter telomeres and an elevated risk of pancreatic cancer [35]. Telomerase activity helps distinguish pancreatic cancer from other pancreatic diseases [97]. Hiyama et al. noted that nearly 95% of pancreatic adenocarcinoma patients exhibited increased TA [98], whereas benign conditions like pancreatitis had lower TA. Testing for telomerase activity in pancreatic juice has proven useful for differentiating malignant intraductal papillary mucinous neoplasms (IPMNs) from benign ones [99]. Additionally, Nakashima et al. found that about 84% of pancreatic ductal adenocarcinomas (PDACs) expressed hTERT [100], and Hashimoto et al. identified increased TA in 83% of invasive ductal adenocarcinomas (IDCs) and hTERT expression in 88% of cases [101]. These findings suggest that hTERT expression might be a more reliable marker than TA alone. Overall, high hTERT levels and increased TA are linked to poorer outcomes in pancreatic cancer [102].

## 3. Telomere Length and Anticancer Therapeutic Strategies

Research has identified several promising therapeutic strategies targeting telomeres and telomerase, which are crucial in maintaining chromosomal stability and preventing genomic instability. In most somatic cells, telomeres progressively shorten with each cell division, eventually leading to cellular senescence and apoptosis. However, more than 85% of cancer cells exhibit increased telomerase activity, which allows cancer cells to avoid senescence [118]. This characteristic makes telomerase a promising candidate for anti-cancer therapies.

Emerging evidence suggests that cancer treatments themselves can influence telomere length. A systematic review of epidemiological studies indicates that the impact of cancer treatment on telomere length varies significantly depending on the type of cancer and treatment regimen [119]. For example, studies on solid tumors have shown significant telomere shortening after chemotherapy, particularly with agents such as doxorubicin, cyclophosphamide, and paclitaxel [119,120]. Conversely, other studies have reported no significant change, or even an increase in telomere length, possibly due to the elimination of cancer cells with shorter telomeres, resulting in a mixed population of normal and malignant cells [119].

One therapeutic strategy involves the use of direct telomerase inhibitors. GRN163L (Imetelstat), a well-studied telomerase inhibitor, functions by binding to the RNA component of telomerase (hTR), thereby inhibiting its activity [121]. Phase II clinical trials have shown that GRN163L can effectively reduce tumor growth and metastatic potential [122]. However, the long-term effects on normal cells that transiently express telomerase, such as germ cells, remain unclear. Another promising approach involves stabilizing G-quadruplex structures within telomeric DNA [122]. These structures can prevent telomerase from accessing telomeres, thereby inhibiting its ability to maintain telomere length [122,123]. This strategy has demonstrated potential in selectively reducing the telomeric 3′ overhang, a critical site for telomerase binding, without affecting overall telomere length, suggesting effective telomerase inhibition [118].

Despite the potential of telomerase inhibition, a subset of cancers—accounting for less than 15%—employ an alternative telomere maintenance mechanism known as the Alternative Lengthening of Telomeres (ALT) [12,30,122,123]. This telomerase-independent pathway enables the maintenance of telomere length through homologous recombination, highlighting the need for therapeutic strategies that can target both telomerase-dependent and ALT-dependent cancers, as these mechanisms contribute to resistance against telomerase-targeted therapies [122]. The TICCA strategy aims to address these challenges by integrating various therapeutic approaches, including telomerase inhibitors, G-quadruplex stabilizers, immunotherapy, and gene therapy, to target multiple components of telomere biology simultaneously [123]. This comprehensive approach is designed to reduce resistance and enhance therapeutic efficacy.

Telomere-mimicking oligonucleotides, known as T-oligos, represent another innovative therapeutic avenue. These short DNA sequences mimic the telomeric 3′ overhang and, when introduced into cancer cells, induce DNA damage responses (DDRs) similar to those triggered by critically shortened telomeres [118,122,124]. This process leads to cell cycle arrest and apoptosis in cancer cells while minimally impacting normal cells. The T11 oligonucleotide (5′-dGTTAGGGTTAG-3′) has shown significant potential in preclinical studies, reducing tumor growth and metastasis with minimal toxicity [122,125,126]. Two models explain the action of T-oligos: the Shelterin Dissociation Model (SDM), which suggests that T-oligos displace the shelterin complex from telomeres, leading to telomere deprotection and DDR activation, and the Exposed Telomere Mimicry Model (ETM), which posits that T-oligos mimic exposed telomeric ends, triggering DDRs similar to those activated by naturally shortened telomeres [118].

While telomerase inhibitors show promise in reducing telomere length and limiting tumor growth, some cancers develop resistance through activation of ALT pathways. Additionally, the long-term effects of these inhibitors on normal, telomerase-expressing cells are not fully understood, underscoring the need for more sophisticated therapeutic strategies. Combining telomerase inhibitors with conventional therapies, such as chemotherapy or radiotherapy, may enhance overall treatment efficacy. Furthermore, novel delivery methods, including cationic polypeptides or lipid nanoparticles, are being explored to improve the stability and effectiveness of T-oligos in vivo [118,127,128,129,130].

Targeting telomere biology remains a promising strategy in cancer therapy, as recent studies have highlighted. Telomere shortening is a hallmark of cellular aging and is closely associated with various chronic diseases, including cancer. Cancer cells often maintain their telomere length through telomerase activation or the ALT mechanism, enabling them to bypass the natural proliferative limits imposed by telomere attrition. Although telomerase inhibition has been explored to limit cancer cell growth [131], this approach can inadvertently activate ALT pathways, complicating treatment outcomes. Moreover, cancer therapies such as chemotherapy and radiotherapy can accelerate telomere shortening in normal cells, raising concerns about therapy-induced aging and long-term health risks for survivors. To address these challenges, developing combination therapies that target both telomerase and ALT pathways, alongside conventional treatments, could provide a more comprehensive strategy for managing diverse cancer types and overcoming resistance [12,30,122,130].

Immunotherapeutic approaches, such as vaccines and adoptive cell therapy, also show promise in targeting telomerase [123]. Additionally, gene therapy aimed at the telomerase gene and promoter is being explored for selectively eliminating cancer cells with high telomerase expression [123,131].

Personalized therapy, utilizing techniques like liquid biopsy and telomere length monitoring to tailor treatments based on individual patient telomere dynamics, could offer a more holistic therapeutic approach [123]. Future research should focus on developing synergistic combinatory therapies, incorporating advanced techniques like CRISPR/Cas9 and oncolytic virotherapies to enhance treatment outcomes and overcome resistance.

All in all, targeting telomeres and telomerase remains a promising avenue in cancer therapy. However, challenges such as resistance mechanisms and the long-term effects of these treatments require further investigation. Developing combination therapies that integrate telomerase inhibitors with standard treatments could provide a more effective strategy for managing a wide range of cancer types and overcoming resistance. Comprehensive, longitudinal studies are needed to better understand the impact of various cancer treatments on telomere length across different cancer types. These studies should employ standardized methodologies and assess telomere dynamics in both peripheral blood and tumor tissues. This would improve our understanding of telomere biology in cancer progression and treatment response, potentially guiding the development of targeted interventions to mitigate the adverse effects of telomere shortening associated with cancer therapies.

## 4. Conclusions

Telomeres, which are repetitive hexameric DNA sequences located at the ends of chromosomes, play a crucial role in safeguarding and replicating the genome. The mechanisms that control telomere maintenance are vital in the progression of cancer, positioning them as key focus areas in cancer treatment research. In many cancers, tumors achieve unlimited replicative potential primarily by activating the enzyme telomerase through increased transcription of the TERT gene. Measuring telomerase activity or hTERT expression in blood may have some diagnostic potential, but issues regarding specificity remain problematic. Activated lymphocytes also show telomerase activity, making lymphocyte contamination a potential confounder. While hTR and hTERT have been studied at the RNA transcript level, a major challenge is the absence of reliable hTERT antibodies for immunohistochemistry. Therefore, targeting telomerase is a prominent strategy in the development of cancer therapeutics.

As telomere biology continues to grow as a research field, there are many avenues for future development to explore. The discovery of better risk biomarkers could allow surveillance and prevention efforts to target high-risk patients specifically, potentially reducing costs, patient anxiety, and the risk of complications significantly.

Although telomere shortening is widely acknowledged as an early marker in cancer development, its role in assessing cancer progression through telomere length and telomerase activity remains controversial. While there is evidence suggesting a connection between telomere length and cancer risk, challenges in study design create uncertainties. Variability in the association between cancer and telomere length further complicates the issue. To better understand gastrointestinal cancer biology, combining telomere length and telomerase activity with clinical factors might be beneficial. This approach could lead to the discovery of new biomarkers, aiding in predicting recurrences and informing treatment decisions, thereby improving clinical management of the disease.

## Figures and Tables

**Table 1:** Studies investigating the association between TL, TA, and gastrointestinal cancer.

Tumor Site	References	
	Telomere Length	Telomerase Activity
Stomach	Wentzensen et al., 2011 [11] Liu et al., 2018 [38] Liu et al., 2009 [49] Hou et al., 2009 [50] Shi et al., 2019 [51] Wang et al., 2018 [52] Cesare et al., 2010 [53] Mushtaq et al., 2022 [54]	Katayama et al., 1999 [55] Rathi et al. [56] Hu et al. [57] Svinareva et al. [58]
Esophagus	Wentzensen et al., 2011 [11] O’Sullivan et al., 2006 [26] Risques et al., 2007 [39] Zeng et al., 2017 [41] Xing et al., 2009 [59] Wennerström et al., 2016 [60] Lv et al., 2017 [61] Li et al., 2020 [62]	Ikeguchi et al. [63] Takubo et al. [64] Mitsui et al. [65]
Colorectum	Pooley et al., 2010 [16] O’Sullivan et al., 2006 [26] Wennerström et al., 2016 [60] Raynaud et al., 2008 [66] Garcia-Aranda et al., 2006 [67] Gertler et al., 2004 [68] Tatsumoto et al., 2000 [69] Engelhardt et al., 1997 [70,71] Hastie et al., 1990 [72] Zee et al., 2009 [73] Li and Lai 2009 [74] Fernández-Marcelo et al., 2016 [75] Rampazzo et al., 2010 [76] Zöchmeister et al., 2018 [77] Peacock et al., 2018 [78] Nikolouzakis et al., 2018 [79] Kroupa et al. [80] Kroupa et al. [81] Ye et al. [82]	Nikolouzakis et al., 2024 [33] Katayama et al., 1999 [55] Garcia-Aranda et al., 2006 [67] Tatsumoto et al., 2000 [69] Engelhardt et al., 1997 [70,71] Bertorelle 2013 [83]
Liver	Tsatsakis et al., 2023 [22] Zeng et al., 2017 [41] Urabe et al., 1996 [84] Cheng et al., 2017 [85] Ma et al., 2017 [86] Rashid et al., 2024 [87]	Park et al., 1998 [88] Nakayama et al., 1998 [89] Satyanarayana et al., 2004 [90] Kobayashi et al., 2001 [91] Kobayashi et al., 2002 [92]
Pancreas	Skinner et al., 2012 [35] Zeng et al., 2017 [41] Ma et al., 2017 [86] Duell 2017 [93] Lynch et al., 2013 [94] Luu et al., 2019 [95] Campa et al., 2014 [96]	Lynch et al., 2013 [94] Luu et al., 2019 [95] Campa et al., 2014 [96] Mizumoto et al., 1996 [97] Hiyama et al., 1997 [98] Inoue et al., 2001 [99] Nakashima et al., 2009 [100] Hashimoto et al., 2008 [101] Kumari et al., 2009 [102]
